# Upregulated WTAP expression in colorectal cancer correlates with tumor site and differentiation

**DOI:** 10.1371/journal.pone.0263749

**Published:** 2022-02-10

**Authors:** Xiao-Fang Dong, Yan Wang, Chih-Hsin Tang, Bi-Fei Huang, Zhang Du, Qian Wang, Jun-Kang Shao, Hua-Jun Lu, Chao-Qun Wang

**Affiliations:** 1 Department of Medical Oncology, Affiliated Dongyang Hospital of Wenzhou Medical University, Dongyang, Zhejiang, China; 2 Graduate Institute of Basic Medical Science, China Medical University, Taichung, Taiwan; 3 Department of Pharmacology, School of Medicine, China Medical University, Taichung, Taiwan; 4 Department of Biotechnology, College of Health Science, Asia University, Taichung, Taiwan; 5 Department of Pathology, Affiliated Dongyang Hospital of Wenzhou Medical University, Dongyang, Zhejiang, China; 6 Department of Anus and Intestine Surgery, Affiliated Dongyang Hospital of Wenzhou Medical University, Dongyang, Zhejiang, China; 7 Department of Oncological Radiotherapy, Affiliated Dongyang Hospital of Wenzhou Medical University, Dongyang, Zhejiang, China; Chung Shan Medical University, TAIWAN

## Abstract

Few reports exist regarding the expression and function of Wilms’ tumor 1-associated protein (WTAP) in colorectal cancer (CRC), and the evidence is controversial. Our analysis explored the expression of WTAP in CRC tissue, and analyzed its clinical and prognostic significance. WTAP expression was significantly higher in CRC tissue than in colorectal adenoma and normal colorectal tissue. WTAP was highest in left colon tumor samples and negatively associated with tumor differentiation, as well as depth of tumor invasion. In multiple logistic regression analysis, independent predictors of WTAP expression in CRC included tumor in the left colon (odds ratio = 2.634; 95% confidence interval: 1.129–6.142; *P* = 0.025) and poorly differentiated tissue (0.072; 0.014–0.367; *P* = 0.002). No clear relationship was observed between CRC patient prognosis and WTAP expression. We suggest that WTAP expression is upregulated in CRC, highly expressed in left colon cancer and negatively correlated with tumor differentiation.

## Introduction

GLOBOCAN 2020 estimates of cancer incidence and mortality ranked colorectal cancer (CRC) as the third most commonly diagnosed cancer worldwide and the second leading cause of death [1]. Wilms’ tumor 1-associated protein (WTAP) is a key component of the human methyltransferase *N*^6^-methyladenosine (m^6^A) complex [2] and is implicated in the initiation and progression of various human cancers [2, 3]. Upregulated levels of WTAP expression facilitate the growth and progression of endometrial cancer via the caveolin-1 (CAV-1)/nuclear factor-κB (NF-κB) axis and are associated with worse survival outcomes [4]. Moreover, WTAP overexpression promotes osteosarcoma tumorigenesis by suppressing homeobox-containing 1 (HMBOX1) expression [5] and is correlated with tumour-associated T lymphocyte infiltration in gastric cancer, indicating a poor prognosis [6]. WTAP also promotes the progression of hepatocellular carcinoma via the HuR-ETS proto-oncogene 1 (ETS1) axis [7], and the upregulation of WTAP in ovarian cancer [8] and bladder cancer [9] is associated with poor prognosis.

WTAP expression and function in CRC is controversial. Evidence suggests that WTAP is an oncogene. For example, the tumour suppressor carbonic anhydrase IV (CA4) inhibits colon cancer development by targeting the WTAP-WT1-TBL1 axis to inhibit Wnt signaling [10]. Furthermore, WTAP is upregulated in CRC tissues compared with normal tissues [11] and is highly expressed in poorly differentiated CRC tissues [12], while oncogene β-arrestin2 (Arrb2) promotes the growth and migration of CRC cells by upregulating WTAP expression [13]. However, in an analysis of records from three databases containing information on associations between human *WTAP* gene expression and CRC prognosis, poor survival was associated with reduced WTAP expression in two databases, while increased WTAP expression was associated with poor survival in the remaining database [14]. Thus, further research is needed to better understand the expression and function of WTAP in CRC.

In this study, we performed immunohistochemical (IHC) analysis to detect WTAP expression in CRC and colorectal adenoma tissue samples obtained from a cohort of Chinese patients. We aimed to clarify the expression of WTAP in CRC and its clinicopathological and prognostic significance.

## Materials and methods

### Patients and tissue samples

Tissue samples were obtained from 375 Chinese patients with CRC (median 69 [range 24–94] years) and 58 patients with colorectal adenoma (median 67 [37–88] years) who underwent primary surgical treatment at the Affiliated Dongyang Hospital of Wenzhou Medical University (Dongyang, Zhejiang, China) between 2008 and 2015. Eighty-one adjacent normal colorectal tissue specimens were obtained from the above-mentioned 81 CRC samples post-surgery. We conducted this study at 2020–2021. During or after data collection, authors can access to information that could identify individual participants. Clinicopathological characteristics were determined for all study participants based on their medical records (see Table 2). Pathohistological and clinical diagnoses satisfied the World Health Organization classification of tumours of the digestive system [15, 16]. CRC patients were staged according to the eighth edition of the American Joint Committee on Cancer (AJCC) Cancer Staging Manual [17]. Follow-up information was available for 258 CRC patients at a median 60 months of follow-up (range 11–60 months). This study was reviewed and approved by the Ethics Committee of the Affiliated Dongyang Hospital of Wenzhou Medical University (2020-YX-063), Dongyang, China. Written informed consent was obtained from all these patients or their guardians. All study methods satisfied the relevant guidelines and regulations issued by the Affiliated Dongyang Hospital of Wenzhou Medical University.

### Tissue array preparation

We followed the methods described by Wang et al., 2020 [18]. The Quick-Ray^®^ UT-06 (Unitma Co., Ltd., Seoul, Korea) tissue microarray system was used to prepare tissue specimens, and we used the Quick-Ray premade recipient block (UB-06) wax model. Three representative sites from each CRC tissue were selected for sampling, and a tissue array with a diameter of 1 mm was made following the manufacturer’s protocol.

### IHC analysis

IHC staining of paraffin-embedded tissue sections used the Envision System (Dako, Glostrup, Denmark), as described previously [19, 20]. The primary antibody was anti-WTAP rabbit monoclonal antibody (clone EPR18744, diluted at 1:3200; Abcam, Cambridge, England). The secondary antibody was Dako’s HRP rabbit/mouse universal antibody (Dako, Glostrup, Denmark). The negative control was incubated with vehicle then with secondary antibody, without primary antibody.

### Assessment of staining

WTAP staining in colorectal tissues was practically uniform throughout all tumor cells, so we needed to only evaluate WTAP staining intensity. The intensity of nuclear staining for WTAP was assessed in CRC tissue and scored on a 4-point scale from 0 (negative) to 1 (weak), 2 (moderate), or 3 (strong) [21–24]. High WTAP expression was defined as a staining intensity of positive invasive cancer cells of 2 or 3 [22, 24]. Each entire section was scanned and scored independently by two pathologists.

### Patient follow-up

We followed the methods described by Wang et al., 2020 [18].

### Statistical analysis

Statistical analyses were conducted using SPSS software version 19.0 (SPSS Inc, Chicago, IL, USA). Between-group differences in WTAP expression were compared using a Pearson’s chi-square test for qualitative variables. Independent correlation factors of WTAP expression were assessed by multivariate logistic regression analysis. Relapse-free survival (RFS) and overall survival (OS) rates were estimated by the Kaplan-Meier method and compared using log-rank testing. *P*<0.05 was considered to be statistically significant.

## Results

### Expression of WTAP in CRC tissue and its relationship with clinicopathological characteristics

WTAP was expressed in the nuclei of CRC cells. The proportion of high WTAP expression in CRC tissue specimens was 80.8% (303/375), compared with 22.4% (13/58) of colorectal adenoma tissues and 19.8% (16/81) of normal colorectal tissue specimens; the expression of WTAP in CRC was significantly higher than that in colorectal adenoma and normal tissue (*P*<0.001) (Table 1).

**Table 1:** WTAP expression in colorectal tissue specimens.

Group	No.	WTAP expression	
		Low expression, n (%)	High expression, n (%)
Normal colorectal	81	65 (80.2%)	16 (19.8%)
Colorectal adenomas	58	45 (77.6%)	13 (22.4%)
Colorectal cancer	375	72 (19.2%)	303 (80.8%)*

* *P*<0.001.

**Fig 1:**
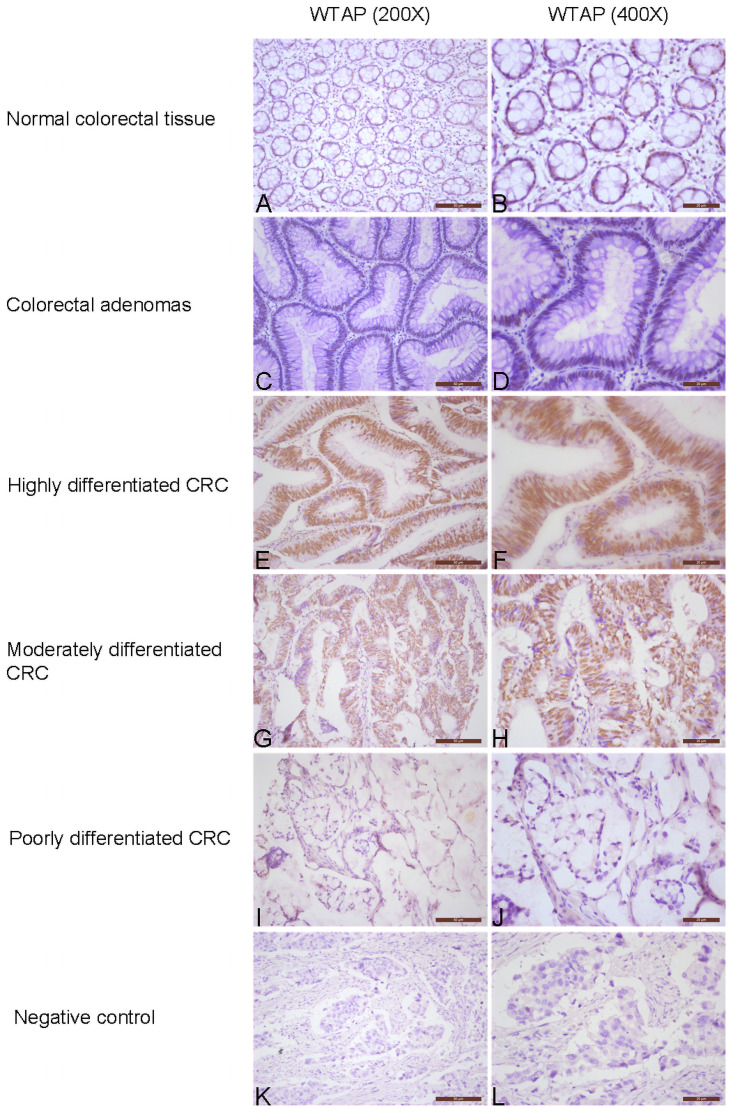
Immunochemical analysis of WTAP expression in colorectal tissue. (A-B) Normal colorectal tissue, low WTAP expression (1+). (C-D) Colorectal adenomas, low WTAP expression (1+). (E-F) Highly differentiated CRC, high WTAP expression (3+). (G-H) Moderately differentiated CRC, high WTAP expression (2+). (I-J) Poorly differentiated CRC, low WTAP expression (0). (K-L) Negative control, nuclear negative.

As shown in Table 2, high WTAP expression was identified in 88.4% (76/86) of left colon cancer tissue, in 81.7% (161/197) of rectal cancer tissue and in 71.7% (66/92) of right colon cancer tissue; the between-group difference was highly significant (*P* = 0.017). The rate of WTAP expression in poorly differentiated CRC tissue (53.5%, 23/43) was significantly lower than that of moderately differentiated (83.0%, 244/294) and highly differentiated CRC tissue (94.7%, 36/38) (*P*<0.001) (Fig 1). In regard to the depth of invasion, the rate of high WTAP expression was significantly lower in the T4 group (75.5%, 123/163) compared with the T3 group (81.9%, 118/144), T2 group (90.9%, 50/55) and Tis+T1 group (92.3%, 12/13) (*P* = 0.049). In logistic regression multivariate analysis, independent predictors of WTAP expression in CRC included left colon tumor site (odds ratio = 2.634; 95% confidence interval: 1.129–6.142; *P* = 0.025) and poorly differentiated tumor (0.072; 0.014–0.367; *P* = 0.002).

**Table 2:** Association of clinicopathological characteristics with high WTAP expression in patients with colorectal cancer.

Variables	No.	High WTAP expression n (%)	*P*-value
**Sex**			
Male	219	177 (80.8%)	0.990
Female	156	126 (80.8%)	
**Age (years)**			
<60	108	81 (75.0%)	0.070
≥60	267	222 (83.1%)	
**Tumor site**			
Right colon	92	66 (71.7%)	0.017
Left colon	86	76 (88.4%)	
Rectum	197	161 (81.7%)	
**Tumor differentiation**			
High	38	36 (94.7%)	<0.001
Moderate	294	244 (83.0%)	
Poor	43	23 (53.5%)	
**Depth of invasion**			
Tis+T1	13	12 (92.3%)	0.049
T2	55	50 (90.9%)	
T3	144	118 (81.9%)	
T4	163	123 (75.5%)	
**Lymph node metastasis**			
No	206	171 (83.0%)	0.230
Yes	169	132 (78.1%)	
**Tumor stage**			
I	49	45 (91.8%)	0.183
II	150	121 (80.7%)	
III	145	113 (77.9%)	
IV	31	24 (77.4%)	

### No association between WTAP expression and survival of patients with CRC

**Fig 2:**
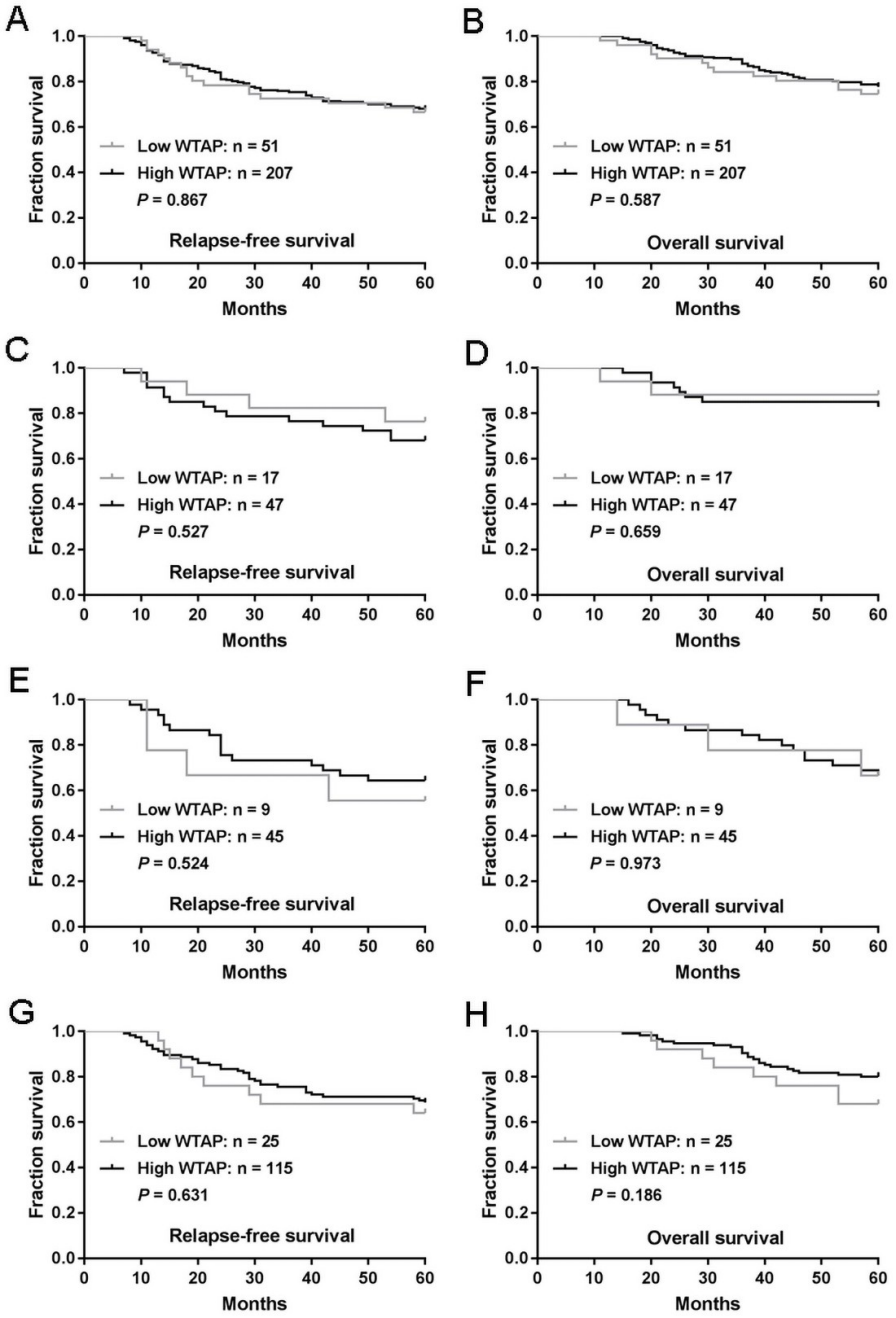
WTAP expression is not associated with the survival of patients with CRC. (A-B) Associations were analyzed between WTAP expression and relapse-free survival (RFS) (A) and also overall survival (OS) (B). (C-D) The associations of WTAP expression with RFS (C) and OS (D) of patients with right colon cancer. (E-F) The associations of WTAP expression with RFS (E) and OS (F) of patients with left colon cancer. (G-H) The associations of WTAP expression with RFS (G) and OS (H) of patients with rectal cancer. *P*-values were calculated using the Mantel-Cox log-rank test.

To assess the potential impact of WTAP expression on survival of CRC patients, we analyzed WTAP expression in relation to RFS and OS rates. Five-year RFS and OS rates were 67.4% and 77.1%, respectively. As shown in Fig 2, no associations were observed between WTAP expression and survival. The 207 patients with high levels of WTAP expression had a mean RFS of 48.8 months and an estimated 5-year RFS rate of 67.6%; corresponding values in the 51 patients whose tumors expressed low levels of WTAP were 48.0 months and 66.7%, respectively (*P* = 0.867; Fig 2A). Mean OS was 54.3 months (with an estimated 5-year OS rate of 77.8%) in the patients with high levels of WTAP expression and 52.9 months (with an estimated 5-year OS rate of 74.5%) in those with low levels of WTAP (*P* = 0.587; Fig 2B).

We analyzed the effect of WTAP expression on the prognosis of right colon cancer, left colon cancer or rectal cancer. As shown in Fig 2C–2H, whether in right colon cancer (Fig 2C and 2D), left colon cancer (Fig 2E and 2F) or rectal cancer (Fig 2G and 2H), the prognosis of tumors that were high WTAP expression did not differ significantly from that of low WTAP group.

## Discussion

Several studies have reported upregulation of WTAP in malignant tumors and the finding that WTAP acts as an oncogene, promoting cancer occurrence and development [4–9]. Results of investigations into the expression and role of WTAP in CRC are controversial. Whereas some studies have shown that WTAP plays an oncogenic role in CRC [10–13], evidence has also shown that reduced WTAP expression is associated with poor survival [14]. Moreover, no research exists regarding the expression and clinical significance of WTAP in large-scale samples of CRC tissue.

Our findings revealed significantly upregulated WTAP expression in CRC tissue compared with colorectal adenoma and normal colorectal tissue, indicating that WTAP may be involved in the occurrence of CRC. Liu *et al* [11] reported finding abundant WTAP expression in colon cancer but not in rectal cancer, but their study only included 22 colon cancer tissue specimens and 21 rectal cancer specimens, and did not further subdivide the left and right colon tumors. In our study, we found that WTAP expression was highest in left colon tumor samples, and the left colon is an independent factor affecting WTAP expression. Evidence shows that tumors arising from right colon, left colon, and rectum each have different biological and molecular features [25–27]. Patients with right colon cancer were more likely to be diagnosed with a more advanced stage and to have more poorly differentiated tumors [25]. Since WTAP expression decreases in higher-stage and poorly differentiated tumors, it makes sense that WTAP expression is lower in right colon cancer. Microsatellite instability-high (MSI-H), deficiency of mismatch repair genes, KRAS and BRAF mutations, and CpG island methylation are more common in right colon cancer than that in left colon cancer [28–32]. Whether the high expression of WTAP in left colon cancer is related to these molecular characteristics needs further study. Wang *et al* [12] detected the expression of WTAP mRNA and they reported finding high WTAP expression in poorly differentiated tissues, but their study was also limited by the small number of samples (10 matched pairs of poorly differentiated and highly differentiated CRC tissue samples). Our study included 375 CRC tissue samples and found that WTAP protein expression negatively correlated with differentiation of the tumor, and that WTAP protein expression is lowest in poorly differentiated tumors. Further analysis showed that poorly differentiated tumors are independently negatively correlated with WTAP expression. We suspect that the expression of WTAP may be inhibited in poorly differentiated CRC; the molecular mechanism needs to be elucidated. Coupled with the established fact that poorly differentiated tumors are associated with higher aggressiveness [15], finding lower WTAP expression in poorly differentiated tumors makes sense that WTAP expression is lower in samples with deeper tumor invasion. According to the above, upregulated WTAP expression in CRC is positively correlated with left colon tumors and negatively correlated with poorly differentiated tumors.

Our survival analysis failed to reveal any association between WTAP expression and survival, which we speculate could be because the oncogenic functions of WTAP are counterbalanced by the suppression of WTAP expression in poorly differentiated tumors. However, in an analysis of records from three databases have shown that poor survival was associated with reduced WTAP expression in two databases, while increased WTAP expression was associated with poor survival in the remaining database [14]. We speculate that these varying results can ascribe to demographic or cohort-specific differences. More research is needed to determine how WTAP exerts oncogenic functions and to clarify the molecular mechanisms underlying its negative correlation with poorly differentiated tumors. Such clarification may enable the manipulation of CRC cell differentiation, and is important for future considerations surrounding the use of WTAP in the inhibition of CRC.

## Supporting information

S1 Table Expression of WTAP in CRC tissue. (XLS)

S2 Table Expression of METTL3/14 in CRC tissue. (XLS)

## Abbreviations

CRC: colorectal cancer
IHC: immunohistochemistry
m^6^A: *N*^6^-methyladenosine
OS: overall survival
RFS: relapse-free survival
WTAP: Wilms tumor 1-associated protein
